# Widening access to perinatal mental health group interventions: learning from a trial of the Circle of Security-Parenting programme in England

**DOI:** 10.3389/fpsyg.2026.1802417

**Published:** 2026-03-26

**Authors:** Zoë Darwin, Jude Field, Lani Richards, Sophia Nahz Rehman, Kavita Trevena, Amy Clarke, Basanti Aryal, Nina Morris, Innamana Pettyll, Ruth O’Shaughnessy, Nic Horley, Kim Alyousefi-van Dijk, Peter Fonagy, Camilla Rosan

**Affiliations:** 1 School of Human and Health Sciences, University of Huddersfield, Huddersfield, United Kingdom; 2 Anna Freud, London, United Kingdom; 3 Cheshire and Merseyside Specialist Perinatal & Maternal Mental Health Services, Mersey Care NHS Foundation Trust, Prescot, United Kingdom; 4 West London Perinatal Mental Health Service, West London NHS Trust, London, United Kingdom; 5 Research Department of Clinical, Educational and Health Psychology, University College London, London, United Kingdom

**Keywords:** accessibility, Circle of Security, groups, inequalities, infant mental health, perinatal mental health, qualitative

## Abstract

**Background:**

Perinatal mental health (PMH) difficulties can adversely affect family relationships across generations. Timely treatment can prevent these impacts, yet inequalities in access persist. Service delivery has also changed rapidly in recent years, including increased provision of online mental health care, creating additional access considerations. This paper offers a framework for widening access to group-based interventions, informed by an analysis of barriers and facilitators to birthing parents accessing the Circle of Security-Parenting (COS-P) programme—an attachment-based group provided by community Perinatal Mental Health Services (PMHS) in England.

**Methods:**

COS-P recipients (birthing parents, typically mothers) and COS-P providers (practitioners) were recruited from a randomised controlled trial involving 10 PMHS in England delivering 51 COS-P groups. The 10-session group intervention was delivered predominantly online, with babies present. Accessibility data were collected via surveys (165 parents), interviews (58 parents and 7 practitioners), and focus groups (6 practitioners). A research team, including co-researchers with lived experience, undertook qualitative content analysis to identify barriers and facilitators to accessing COS-P and to explore how these operated across different aspects of the intervention format. Recommendations were co-produced with a lived-experience panel to inform how access might be widened.

**Results:**

We developed an access framework conceptualising access as a pathway involving four steps: attend, take part, understand, and apply. This enabled examination of how 13 barriers and facilitators manifested in relation to each aspect of the intervention format, and how they influenced pathway steps individually, cumulatively, or in opposing ways. Features that supported attendance sometimes constrained opportunities to take part, understand, or apply learning. The influence of barriers and facilitators also varied by individual and family context, and by location context—defined as where access happens (community venues, homes, online spaces) and when it happens (during or between sessions).

**Conclusion:**

Recognising the complexity of accessibility may support efforts to widen access in ways that are more meaningful to individuals and families. These findings extend beyond COS-P, offering a framework to improve equitable access to group-based psychological interventions in the perinatal period and helping ensure that seemingly convenient ways of working do not inadvertently perpetuate inequalities.

## 1 Introduction

Perinatal mental health (PMH) difficulties are common worldwide and, as highlighted by the World Health Organization, evidence-based early identification and management is essential (Howard and Khalifeh, 2020). If left untreated, PMH difficulties can negatively affect parents, children, and wider family functioning (Bauer et al., 2014). Although the relationship between PMH difficulties and adverse child developmental outcomes is complex and multi-factorial, research consistently identifies the quality of the parent-infant relationship as a key pathway of influence, whereby symptoms may constrain a parent’s capacity for sensitive, responsive and attuned caregiving (Stein et al., 2014). These difficulties can be experienced by birthing parents (i.e., mothers, and trans and gender diverse birthing parents) and non-birthing parents (i.e., fathers and other co-parents). While some countries have practitioners specifically trained to deliver perinatal care, England is distinctive in having nationwide specialist community-based PMH provision for birthing parents with moderate-to-severe and complex mental health difficulties requiring more intensive support than can be provided in primary care (Gurol-Urganci et al., 2024). Recent policy (NHS Mental Health Implementation Plan 2019/20–2023/24) sets out an ambition to increase access to evidence-based perinatal psychological therapies for birthing parents, parent-infant dyads, and couples and families (NHS England, 2019). However, NHS PMHS remain primarily oriented towards maternal mental health, and interventions explicitly targeting parent-infant attachment and couple and family relationships are less common or embedded, highlighting a persistent gap in meeting the broader relational needs of families affected by PMH difficulties.

Although PMHS are commissioned based on an estimated 10% prevalence of birthing mothers requiring specialist support (Fisher et al., 2024), concerns remain about barriers and facilitators to accessing PMH care (Webb et al., 2023). Importantly, these barriers and facilitators are not experienced uniformly: inequalities are evident across identification of need in universal services, referral, and uptake of specialist support. These disparities disproportionately affect people from racially and ethnically minoritised communities, those who are not fluent in English, and those from socioeconomically deprived backgrounds (Darwin et al., 2022; Jankovic et al., 2020; Prady et al., 2021). Data are not available on access rates by treatment type within PMHS; however, it is plausible that group-based programmes may be differentially accessed by marginalised groups, given international evidence of racial and ethnic disparities in access to community-based PMH programmes (Rokicki et al., 2024).

The pandemic accelerated rapid innovation, including increased remote delivery of care – including group interventions—often without a clear understanding of implications for accessibility. Emerging evidence suggests that synchronous remote online delivery may improve access and retention by reducing financial costs, travel burden, and childcare requirements (Iacob et al., 2025), yet concerns persist regarding digital poverty and exclusion (Jovanović et al., 2025).

Alongside evaluating clinical effectiveness, it is essential to explicitly consider accessibility and inequalities in access to the intervention in question as part of formal intervention evaluation. This paper reports findings from a process evaluation conducted within a clinical and cost-effectiveness trial (the COSI trial; Rosan et al., 2023) evaluating the 10-session COS-P group programme (Powell et al., 2013). Further detail is available in the trial protocol (Rosan et al., 2023) and the clinical effectiveness publication (Rosan et al., 2025). The process evaluation aimed to examine the accessibility of COS-P when delivered in NHS PMHS, by identifying barriers and facilitators from the perspectives of parents (group recipients) and practitioners (group providers).

## 2 Methods

### 2.1 Ethics approval and consent to participate

Ethical approval was obtained on 26/11/2021 from the Surrey Research Ethics Committee (REC ref.: 21/LO/0723). The study adhered to the Declaration of Helsinki. Written, informed consent was obtained from all participants.

### 2.2 Recruitment processes and sample characteristics

Participants were recruited from the intervention arm of the COSI trial, delivered across 10 PMHS in England and comprising 51 COS-P groups (Rosan et al., 2025). Recruitment procedures and study materials were co-developed with the trial’s lived experience panel and are described in the trial protocol (Rosan et al., 2023). For the purposes of this study, (parent) participants allocated to COS-P were classified as non-starters (did not begin a group), non-completers (attended 1–5 sessions), or completers (attended ≥6 sessions, defined as a clinical dose). The mean number of sessions attended was 6.4 (median = 8).

**Figure 1:**
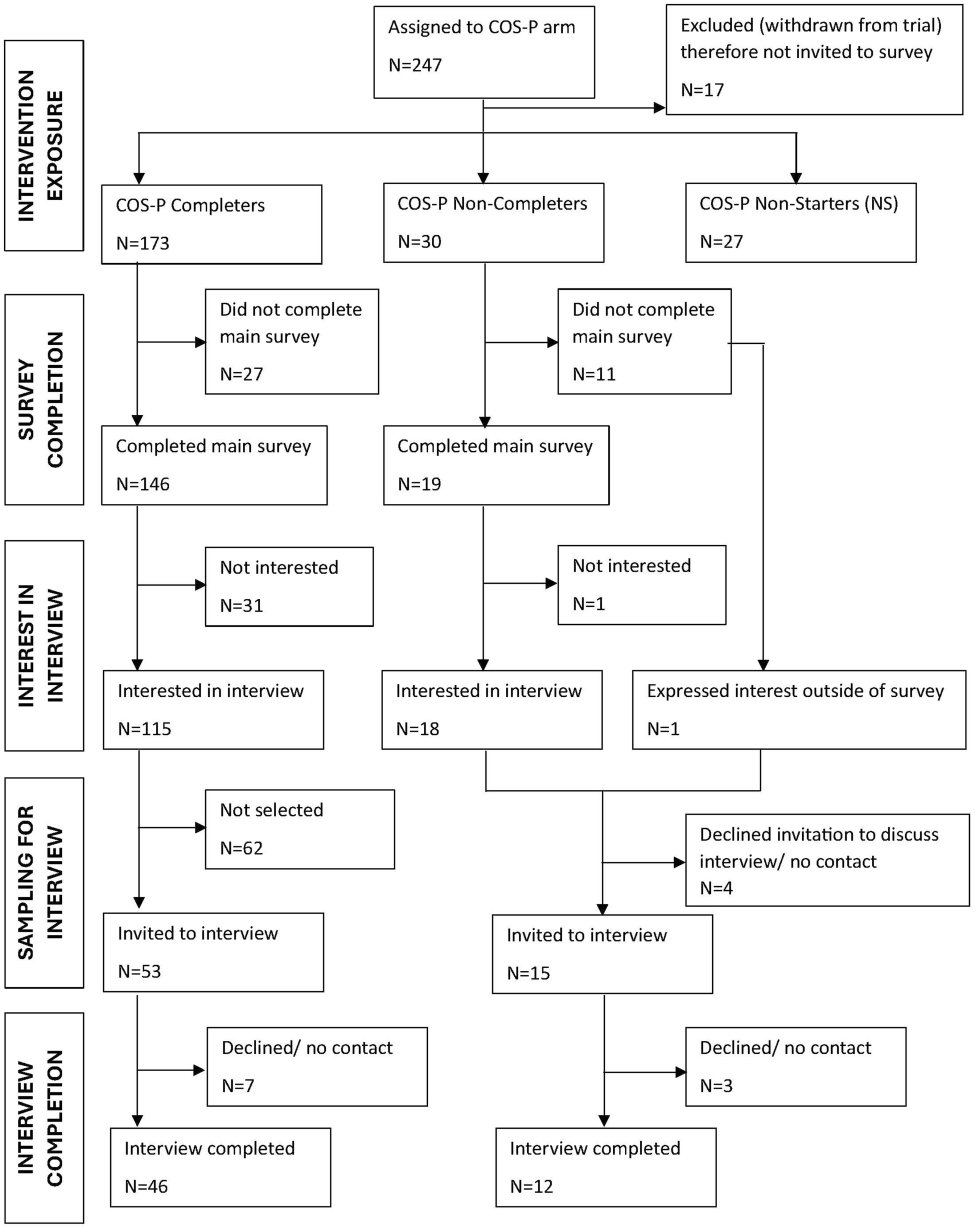
Parent participant flow.

All COS-P completers (*n* = 173) and non-completers (*n* = 30) who remained active in the trial at three-month follow-up were invited to complete an accessibility survey. To maximise inclusion, invitations were issued by email and telephone, and participants could opt for self-completion or assisted completion. The survey response rate was 81.3% (165/203; Figure 1), with ≥75% response at 9 of the 10 sites. Respondent characteristics were comparable to the invited sample (Table 1). Pre-intervention psychological symptoms and trauma histories (CORE-OM: Evans et al., 2002; PBQ: Brockington et al., 2006; CTQ: Bernstein et al., 2003) were similar for survey respondents and those subsequently interviewed.

**Table 1:** Parent sample characteristics.

Characteristic	Invited to main survey (*N* = 203)	Completed main survey (*N* = 165)	Completed interview (*N* = 58)
Age (years)	31.1 (5.2)	31.5 (5.1)	31.9 (5.0)
Ethnicity			
White British	167 (82%)	137 (83%)	43 (74%)
White other	13 (6%)	10 (6%)	7 (12%)
Other	9 (4%)	9 (5%)	5 (9%)
Missing	14 (7%)	9 (5%)	3 (5%)
Relationship status			
Single	17 (8%)	14 (8%)	5 (9%)
In a relationship (living together)	170 (84%)	140 (85%)	48 (83%)
In a relationship (not living together)	3 (1%)	3 (2%)	1 (2%)
Not known	2 (1%)	2 (1%)	2 (3%)
Missing	11 (5%)	6 (4%)	2 (3%)
Sexual orientation			
Straight	173 (85%)	144 (87%)	47 (81%)
Other	25 (12%)	18 (11%)	10 (17%)
Missing	5 (2%)	3 (2%)	1 (2%)
Gender			
Woman	186 (92%)	155 (94%)	52 (90%)
Man (including trans man)	0 (0%)	0 (0%)	0 (0%)
Non-binary	4 (2%)	3 (2%)	3 (5%)
Other	1 (<1%)	1 (1%)	1 (2%)
Missing	12 (6%)	6 (4%)	2 (3%)
Mental health difficulties leading to referral to PMHS:			
Depression			
No	32 (16%)	24 (15%)	10 (17%)
Yes	171 (84%)	141 (85%)	48 (83%)
OCD			
No	174 (86%)	144 (87%)	50 (86%)
Yes	29 (14%)	21 (13%)	8 (14%)
Anxiety			
No	33 (16%)	25 (15%)	6 (10%)
Yes	170 (84%)	140 (85%)	52 (90%)
Personality difficulties			
No	172 (85%)	141 (85%)	52 (90%)
Yes	31 (15%)	24 (15%)	6 (10%)
Trauma			
No	116 (57%)	98 (59%)	34 (59%)
Yes	87 (43%)	67 (41%)	24 (41%)
Psychosis			
No	196 (97%)	160 (97%)	54 (93%)
Yes	7 (3%)	5 (3%)	4 (7%)
Bi-polar disorder			
No	195 (96%)	160 (97%)	56 (97%)
Yes	8 (4%)	5 (3%)	2 (3%)
Other			
No	193 (95%)	157 (95%)	54 (93%)
Yes	10 (5%)	8 (5%)	4 (7%)
Previous mental health difficulties			
Yes	178 (88%)	145 (88%)	55 (95%)
No	17 (8%)	15 (9%)	2 (3%)
Prefer not to say	3 (1%)	2 (1%)	0 (0%)
Missing	5 (2%)	3 (2%)	1 (2%)
Income above deprivation threshold			
Yes	96 (47%)	83 (50%)	22 (38%)
No	97 (48%)	76 (46%)	34 (59%)
Missing	10 (5%)	6 (4%)	2 (3%)
Highest completed level of education			
Primary education or less	2 (1%)	2 (1%)	1 (2%)
Secondary education	12 (6%)	9 (5%)	5 (9%)
Tertiary/further education (e.g., college)	60 (30%)	47 (28%)	18 (31%)
Higher education (e.g., University degree)	115 (57%)	98 (59%)	31 (53%)
Other general education	2 (1%)	2 (1%)	1 (2%)
Prefer not to say	1 (<1%)	1 (1%)	0 (0%)
Missing	11 (5%)	6 (4%)	2 (3%)
Housing			
Homeowner	115 (57%)	102 (62%)	33 (57%)
Other	74 (36%)	56 (34%)	22 (38%)
Missing	14 (7%)	7 (4%)	3 (5%)
Employment status			
Employed or self-employed	135 (66%)	113 (68%)	39 (67%)
Unemployed or in education/training	52 (26%)	42 (25%)	17 (29%)
Missing	16 (8%)	10 (6%)	2 (3%)
Religion			
Christian	66 (33%)	56 (34%)	19 (33%)
None	118 (58%)	94 (57%)	31 (53%)
Other	7 (3%)	6 (4%)	2 (3%)
Prefer not to say	7 (3%)	6 (4%)	5 (9%)
Missing	5 (2%)	3 (2%)	1 (2%)
Country of birth			
United Kingdom	167 (82%)	139 (84%)	47 (81%)
Elsewhere	13 (6%)	11 (7%)	7 (12%)
Missing	23 (11%)	15 (9%)	4 (7%)
First language			
English	184 (91%)	151 (92%)	50 (86%)
Other language (but with good knowledge of English)	8 (4%)	8 (5%)	6 (10%)
Missing	11 (5%)	6 (4%)	2 (3%)
Child age (in weeks)	21.0 (12.2)	20.8 (12.4)	20.8 (12.6)
Child first born status (measured as having more than one <18 year old in the household)			
Not first born	102 (50%)	77 (47%)	34 (59%)
First born	101 (50%)	88 (53%)	24 (41%)
Number of previous pregnancies			
0	69 (34%)	58 (35%)	17 (29%)
1	53 (26%)	41 (25%)	11 (19%)
>1	81 (40%)	66 (40%)	30 (52%)
Child sex			
Female	96 (47%)	80 (48%)	31 (53%)
Male	103 (51%)	83 (50%)	26 (45%)
Missing	4 (2%)	2 (1%)	1 (2%)
CTQ score at baseline	48.6 (20.1)	47.9 (20.6)	48.1 (20.2)
CORE-OM score at baseline	64.9 (20.5)	63.7 (20.3)	67.7 (20.6)
CORE-OM score at 3 m	52.5 (20.3)	52.5 (19.5)	56.7 (18.2)
PBQ score at baseline	34.2 (16.6)	34.5 (15.8)	37.8 (17.3)
PBQ score at 3 m	24.6 (14.2)	25.0 (13.3)	27.0 (14.0)

mean (sd) for continuous variables, *n* (%) for categorical variables.

Interview invitations were offered to all non-completers and to a purposive subsample of completers. Maximum variation sampling (Sandelowski, 1995) was used to guide interview invitations, aiming for diversity across background characteristics (e.g., ethnicity, relationship status, parity, infant age), site, recruitment block, sessions attended, and group size. Additional interviews were conducted with completers who had missed multiple sessions to strengthen understanding of access challenges. In total, 58 parent interviews were completed (Figure 1; Table 1).

All practitioners who facilitated at least one COS-P group (*n* = 21) were eligible to participate in an interview or focus group. Nineteen were contactable and 13 participated (7 interview participants; 6 focus group participants). Practitioner roles included Practitioner Psychologists (*n* = 10) and Mental Health Nurses (*n* = 3). All received COS-P training for the trial, were encouraged to run a practice group, and were offered 20 h of supervisory coaching from COS-P developers. Most practitioners (11/13) had prior clinical experience of parent-infant work, and 12/13 had prior group facilitation experience; however, only four had facilitated online groups prior to the trial.

### 2.3 Data collection materials

Data collection materials, co-developed with the lived experience panel, explored barriers and facilitators to accessing COS-P, focusing on both the intervention delivery format and wider contextual influences.

The survey combined closed questions organised around nine COS-P format elements as shown in Table 2: (1) COS-P resources (session content and workbook content); (2) practitioner role (lead facilitator and co-facilitator); (3) peer group format; (4) group size; (5) structure of group sessions (10 two-hour sessions); (6) modality (predominantly online with up to two in-person sessions); (7) presence of babies (here, welcome to attend); (8) presence of older children (not able to attend); and (9) presence of parenting partners (i.e., people who provide parenting-related support not able to attend, with attendance of adults restricted to birthing parents under the care of the service and any interpreters). Each of these was accompanied by an open-ended question where participants could provide further comment. The full survey content, including additional questions not focused on access, is available in the published protocol (Rosan et al., 2023).

**Table 2:** Survey ratings concerning accessibility in relation to different aspects of the COS-P intervention format (*n* = 165 respondents).

Question	Response	Frequency (%)
*Session length* Each session lasted about 90 min. Did you find the length of each session:	Too long	38 (23.0)
	About right	119 (72.1)
	Too short	8 (4.8)
*Group size* Did you find the size of your group:	Too many	1 (0.6)
	About right	134 (81.2)
	Too small	30 (18.2)
*Helpfulness of other parents* How helpful did you find it to be in a group with other parents?	Very unhelpful	0 (0)
	Unhelpful	2 (1.2)
	Neither helpful or unhelpful	14 (8.5)
	Helpful	50 (30.3)
	Very helpful	97 (58.8)
	Missing	2 (1.2)
*Helpfulness of facilitator* How helpful did you find the group facilitator(s)?	Very unhelpful	0 (0)
	Unhelpful	0 (0)
	Neither helpful or unhelpful	6 (3.6)
	Helpful	32 (19.4)
	Very helpful	125 (75.8)
	Missing	2 (1.2)
*Helpfulness of materials and ideas* Considering your baby’s age, how helpful did you find the material and ideas covered?	Very unhelpful	0 (0)
	Unhelpful	5 (3.0)
	Neither helpful or unhelpful	9 (5.5)
	Helpful	83 (50.3)
	Very helpful	66 (40.0)
	Missing	2 (1.2)
*Modality preferences* If there was an option to access the group online or face-to-face in-person, which would you prefer?	Online	34 (20.6)
	Face-to-face	49 (29.7)
	No preference	5 (3.0)
	Combination	71 (43.0)
	Not sure	6 (3.6)
*Babies’ presence preferences* Would you prefer having the option for babies to be present at the group?	Not to have babies attend sessions	22 (13.3)
	To have babies attend all the sessions	76 (46.1)
	Prefer to have babies attend some of the session	36 (21.8)
	Not sure	31 (18.8)
*Parenting support preferences* Would you prefer having the option for a person supporting you in parenting (e.g., partner, parent, close friends) to attend the group?	Yes	65 (39.4)
	No	58 (35.2)
	Not sure	42 (25.5)

Interviews explored intervention accessibility for two additional elements not covered in the survey, relating to structures provided by PMHS outside the group sessions: (10) pre-group contact with practitioners and (11) catch-up sessions (where group sessions were missed). Lived experience consultation identified these as more suitable for qualitative exploration via interview than survey. Interviews also enabled clarification of survey responses and elaboration of experiences. Practitioner interviews and focus groups explored barriers and facilitators to access, with explicit consideration of contextual factors. All data collection materials are available in the protocol (Rosan et al., 2023).

### 2.4 The intervention

COS-P is a manualised, attachment-based programme drawing on psycho-educational, cognitive-behavioural and psychodynamic principles. It is organised into eight modules and uses facilitator-led video review, visual materials, and guided reflection to support parents to recognise and respond to their own and their child’s emotional needs (Powell et al., 2013).

As outlined in the COSI trial protocol (Rosan et al., 2023), groups used a perinatal adaptation of COS-P, facilitated by a trained lead practitioner and a co-facilitator without formal COS-P training. The adaptation extended delivery to 10 sessions, added prompts to support discussion of concerns specific to parents of very young infants, and incorporated baby-focused images into key diagrams used in the resources.

### 2.5 Analysis

Analysis used qualitative content analysis, combining inductive and deductive elements (Elo and Kyngäs, 2008; Vaismoradi et al., 2016). The main analysis team comprised academic researchers (ZD, JF) and co-researchers (LR, SNR, KT, AC), hereafter, referred to as lived experience co-researchers. These co-researchers were parents with lived experience of being cared for by PMHS, bringing a range of experiences across different types of difficulties, geographical areas within England, and diverse work backgrounds, with most being new to qualitative data analysis.

For initial coding, academic researchers (ZD, JF) independently undertook open coding of free-text survey responses and relevant interview and focus group data. Coding was inductive, allowing codes and early category ideas to be generated from the data, rather than using an *a priori* coding structure. The analysis also inevitably had deductive aspects, in being guided by the study aim (barriers and facilitators to access) and in having the survey and interview materials organised around intervention-format areas. To minimise the potential for deductive elements to constrain inductive insights, codes were created whenever new insights emerged; data-driven ideas were valued, as were discussions of different perspectives amongst researchers.

Codes were organised into higher-order categories (Vaismoradi et al., 2016), representing empirically derived barriers and facilitators to access. Descriptive summaries of closed-question survey data were used to contextualise qualitative patterns. Categories and working definitions were iteratively discussed and refined by an analysis subgroup (ZD, JF), then reviewed and further refined by the full qualitative team across a series of meetings. In preparation for these meetings, lived experience co-researchers (LR, SNR, KT, AC) completed analysis activities with data excerpts, including independently assigning codes and categories (using proposed categories where helpful, but not limited to these) and scrutinising categories applied by others (ZD, JF). This involvement offered checks against deductively driven over-interpretation, through challenging category boundaries and discussing alternative groupings, helping ensure that inductively generated insights were retained. This approach supported collaborative strengthening of the analysis and was well suited to a team combining researchers with varied qualitative experience and co-researchers undertaking qualitative analysis largely for the first time.

Team members (ZD, BA, NM, IP) undertook further coding of survey data and relevant transcript sections using NVivo, independently applying and testing the codes and categories, and refining these through a series of meetings, leading to minor adjustments. The final analytic framework was reviewed through additional online meetings with the qualitative team (ZD, JF, LR, SNR, KT, AC) and clinical members of the trial research team (CR, ROS, NH).

Drawing on Krippendorff (2018) and Lindgren et al. (2020), we undertook a structured process of abstraction to move beyond descriptive categories and identify broader interpretive patterns. This involved examining relationships among categories and exploring how these patterns related to COS-P delivery format and parents’ broader contexts. Discussions were also informed by reflections from those who conducted the interviews (JF) and focus groups (JF, ZD, LR), and by lived experience perspectives, including those with and without personal experience of COS-P. Through iterative team discussions, including an in-person mapping exercise aligning categories with COS-P format areas and emergent elements of access (Supplementary file S1), we generated two overarching analytical contributions. First, we conceptualised access as a four-step pathway (attend, take part, understand, apply). Second, we identified contextual factors (individual, family, location) shaping how barriers and facilitators were experienced by parents, and how intervention format could interact with these contexts.

### 2.6 Involvement of the wider lived experience panel

The wider lived experience panel reviewed descriptive survey findings and sense-checked our developing framework, helping to enhance its face validity and practical relevance. A dedicated panel meeting was used to co-produce practical considerations intended to support accessibility for each intervention-format area relating to the group sessions. These co-produced lived experience panel recommendations are presented within the results (Section 3.3).

### 2.7 Reflexive statement

Among the lived experience co-researchers, one member had received COS-P in person, and others had received group-based psychological interventions within PMHS, including online provision. The academic qualitative researchers include birthing and non-birthing parents. One lived experience co-researcher has shadowed COS-P in a professional role, although most of the formal analysis pre-dated this experience. Practitioners within the wider study team include individuals with experience of in-person and online delivery of group-based psychological interventions in this clinical context, including COS-P. The team includes some cultural and linguistic diversity, and members with clinical and academic interests in family-inclusive ways of working. We also note potential parallels with our own experience of conducting the trial largely online, alongside some in-person meetings.

## 3 Results

These findings are informed by analysis of data collected from 165 parents who completed the accessibility survey (81.3% response rate), including 58 who also took part in interviews, and from 13 practitioners (seven interview participants and six focus group participants).

### 3.1 Conceptualising access as a pathway

**Figure 2:**
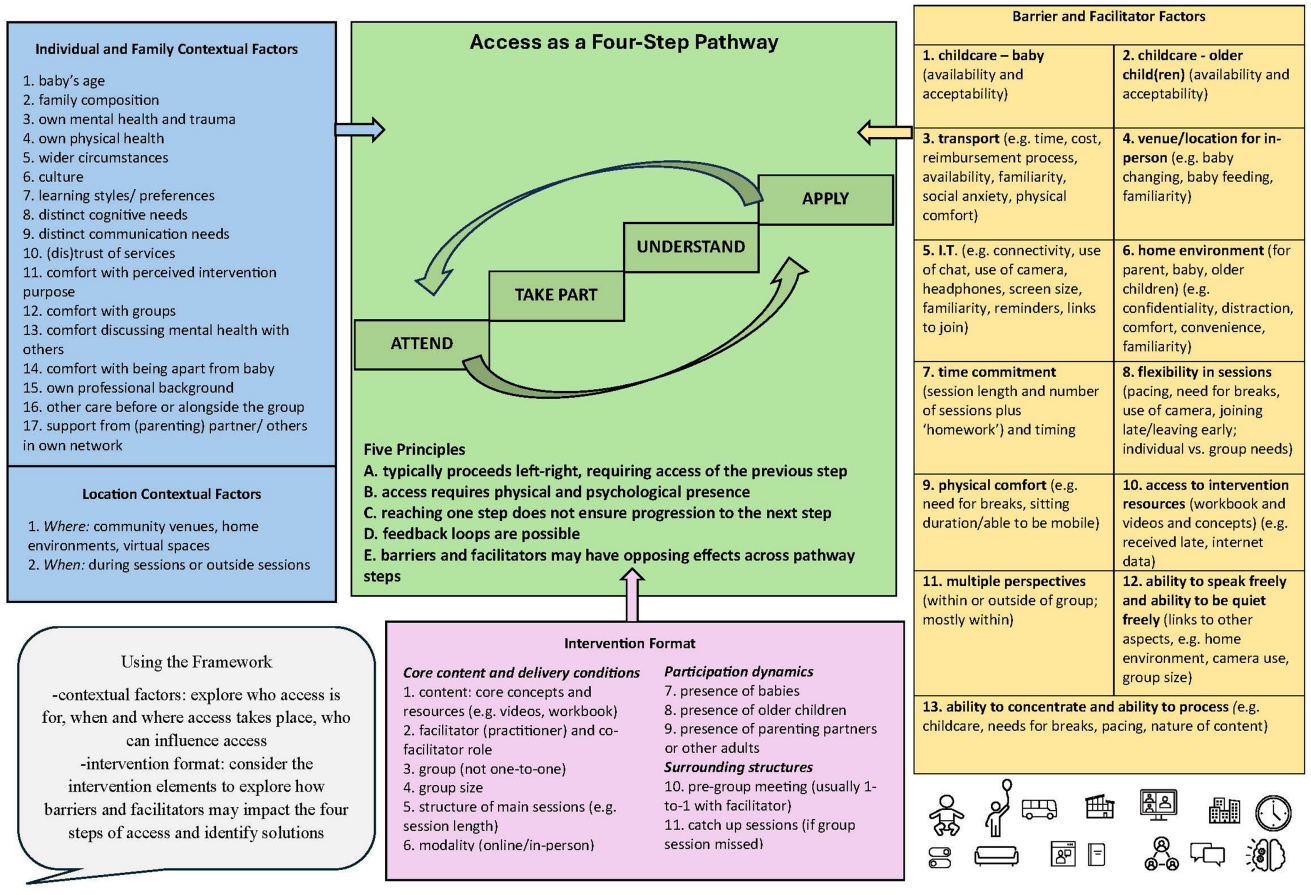
Framework for access to group-based psychological interventions in perinatal mental health care.

Our analysis conceptualised access as a pathway involving four steps: (i) attend, (ii) take part, (iii) understand, and (iv) apply the learning. As is visually depicted in Figure 2 (central green section), the pathway is shaped by barriers and facilitators (hereafter, “b/f”; right-hand yellow section) and contextual factors (individual, family, location; left-hand blue section). The intervention-format areas (lower central pink section) provide a structured way of seeing how these influences play out across the access pathway. To support clarity in Figure 2 and cross-referencing with the main text, items within each coloured section are numbered only for ease of reference; these numbers do not indicate hierarchy.

Based on our analysis, the pathway is characterised by five principles that describe how it may function:

**Principle A: Access typically proceeds left–right**. Earlier steps generally enable later ones. For example, a factor compromising taking part (step ii) may reduce capacity to understand (step iii) and apply (step iv), as seen with small screen size (b/f 5) or a disruptive home environment (b/f 6). Barriers and facilitators may therefore have cumulative effects across steps.

**Principle B: Access requires both physical and psychological presence**. Although access is often assessed through physical presence—captured as attendance (step i), whether in person or remote—parents’ concerns commonly related to psychological access across steps ii–iv. Some barriers and facilitators were predominantly physical (e.g., transport, b/f 3), others predominantly psychological (e.g., ability to speak or be quiet freely, b/f 12), and others spanned both domains (e.g., flexibility in sessions, b/f 8, with downstream links to physical comfort, b/f 9, and cognitive processes, b/f 13). Access was not necessarily observable to others. Some parents described being mentally absent (e.g., due to trauma-related triggers) while attending sessions; conversely, others could be perceived as “absent” because they chose not to use a camera, speak, or contribute by writing, while nevertheless taking part and understanding.

**Principle C: Reaching one step does not ensure progression to the next**. A factor may facilitate a single step without influencing others. For example, reliable transport (b/f 3) supports attendance (step i) but may not promote taking part (step ii); multiple perspectives within a group (b/f 11) may support understanding (step iii) but not application where contextual constraints limit implementation (step iv).

**Principle D: Feedback loops are possible**. Facilitators of later steps may strengthen earlier steps. For example, applying learning (step iv) may deepen understanding (step iii) or increase willingness to take part (step ii), which in turn can support continued attendance (step i).

**Principle E: Barriers and facilitators may have opposing effects across steps**. A single factor may be access-enabling for one step while being access-constraining for another. For example, childcare provision (b/f 1) may support attendance (step i) but reduce understanding (step iii) if the parent cannot observe their baby during the group. Likewise, allowing babies to attend (format area 7) may enable attendance (e.g., by removing childcare need or reducing time burden, b/f 7) yet hinder taking part, understanding, and application (steps ii-iv) due to distraction (b/f 13) or reduced ability to speak freely (b/f 12).

### 3.2 Contextual factors

Our analysis highlighted the importance of contextual factors in shaping how barriers and facilitators were experienced and how these interacted with intervention format. Contextual factors were grouped into location contextual factors and individual and family contextual factors (Figure 2, blue section). These factors did not operate independently of barriers and facilitators; rather, they shaped their relevance, influence, and direction of effect across the four pathway steps.

#### 3.2.1 Location contextual factors—where and when access takes place

Location contextual factors were categorised to describe where and when access takes place: (1) in-person community venues, homes, and virtual spaces; and (2) during sessions and outside sessions. These do not map directly onto all 13 barriers and facilitators (b/f) but provide a lens for understanding how access is shaped by context.

**Where: In-person community venues, homes, and virtual spaces**. Barriers and facilitators varied across these settings. Some were particularly salient for in-person access in community venues (e.g., transport, b/f 3; venue/location, b/f 4). For remote participation, analysis indicated the need to conceptualise access as involving two locations: home environments and virtual spaces. Virtual spaces were constituted through features such as screen “boxes” (where each member appears in their own frame), blurred backgrounds, audio muting, and chat functions. Some parents described the end of a session as involving a transition from a virtual space back into their home surroundings. Certain factors were specific to remote access in homes (home environment, b/f 6), others were relevant to both homes and virtual spaces (e.g., I. T., b/f 5), and some cut across all three locations (e.g., flexibility in sessions, b/f 8).

**When: During and outside sessions**. While most barriers and facilitators were experienced during COS-P sessions, some also operated outside sessions. For example, time commitment (b/f 7) included not only the travel time required for attendance but also the time needed to complete “homework” to support understanding and application, which in turn had knock-on effects on parents’ ability to take part in subsequent sessions. Access to COS-P resources between sessions (b/f 10) shaped understanding and application, including parents’ ability to share learning with—and receive support from—partners or other family members. Multiple perspectives (b/f 11) primarily promoted understanding through group discussion, but could also be encountered outside sessions (e.g., through family members’ perspectives).

#### 3.2.2 Individual and family contextual factors—who access is for and who can influence access

Individual contextual factors (Figure 2) included parents’ mental health and trauma, physical health, cognitive, communication, or processing needs, and comfort with particular aspects of the intervention and its format. These shaped access across the four steps and could intensify or mitigate the relevance of specific barriers and facilitators.

Family contextual factors included infant age, family composition (including perinatal loss and older children), and the presence or absence of supportive others involved in parenting, such as partners, ex-partners, or extended family. These factors were relevant both to who access applied to and to who else could influence parents’ ability to attend, take part, understand, or apply learning. For example, a supportive partner could strengthen understanding and application, whereas unsupportive dynamics could constrain these steps.

Together, these individual and family contextual factors shaped not only parent and infant access during sessions but also the involvement of babies, older children, and other adults in understanding or applying COS-P principles outside sessions. Further examples are provided in Supplementary file S2.

### 3.3 Barriers and facilitators in relation to intervention-format areas with co-produced recommendations

In this section we illustrate, format area by format area, how barriers and facilitators affect each pathway step, noting where effects oppose each other and where context shifts the balance. Sections 3.3.1–3.3.9 concern format elements relating to group sessions and include key survey descriptives (reported fully in Table 2), alongside co-produced recommendations from the lived experience panel. The summaries in Section 3.3.10 (pre-group contact) and Section 3.3.11 (catch-up sessions) were not assessed in the survey or included in the panel consultation and therefore draw only on interview and focus group data.

Supplementary file S1 (mapping exercise) indicates that some barriers and facilitators were more closely linked to particular aspects of the intervention format. For example, transport (b/f 3) aligned closely with modality (online/in-person; format area 6) and the option for babies to be present (format area 7). Conversely, other barriers and facilitators—such as ability to speak freely and ability to be quiet freely (b/f 12)—were relevant across most or all format areas. In the main text, we present example barriers and facilitators for each format area alongside selected illustrative quotes to reflect variation in experiences and, where relevant, to highlight contextual factors shaping their influence. Participant identifiers accompanying quotes indicate participant type (parent or practitioner). Parent identifiers additionally indicate study site (A–J) and number of group sessions and catch-up sessions accessed, including—where applicable—whether fewer than 10 sessions were offered because content was completed within nine sessions (e.g., “7+2; 9 sessions offered” denotes seven group sessions and two catch-up sessions out of a possible nine).

#### 3.3.1 Content: concepts and materials

Half of respondents (50.3%, 83/165) found the COS-P concepts and materials helpful for their baby’s age, with a further 40.0% (66/165) rating them as very helpful. Parents valued the materials for supporting understanding (step iii) and application (step iv). Family context shaped the feasibility of applying ideas (step iv). A small number of accounts indicated potential distress and self-blame where parents experienced the timing of the intervention as “too late” for their personal context, either in relation to their baby or older children. More commonly, parents and practitioners felt the materials were better suited to older infants and children. For some, this reduced perceived relevance; others framed the content as supporting future application with their baby rather than being closely aligned to their baby’s current developmental stage. This illustrates how developmental mismatch can reduce parents’ ability to visualise examples, undermining understanding (step iii) and later application (step iv).

“*Challenging as [baby] was only 4 or 5 months old so difficult to visualise and experience examples*.” (G30; 10+0)

“*It would be really interesting to do it again when [baby]’s a bit older. I’d like to, as a refresher type thing. Because a lot of the mums had already had children, so they were doing it for, not necessarily their babies, but for their older children too, whereas I’m having to remember everything that I learned for [baby] when she gets older*.” (A14; 10+0)

A small number of participants described challenges applying learning (step iv) as a single caregiver responsible for multiple children, indicating the role of contextual constraints.

“*I didn’t agree with some of the content. I thought a lot of ideas were not as easy to actually put into practice in real life. … There was a lot of times where the theory just would not work in my situation because I’m a single parent and because I’m a single parent to two children of very different ages*.” (D12; 10+0)

More commonly, parents described difficulty applying learning consistently in day-to-day life. While some reported that increased understanding of babies’ communication and needs helped them to slow down, notice cues, and interrupt negative thought spirals, others experienced COS-P as not sufficiently supporting self-regulation skills, limiting sustained application (step iv).

Many participants praised the COS-P resources for making complex ideas accessible (i.e., taking part, step ii, and understanding, step iii). A minority, however, reported struggling with the complexity of the language used and processing load, compromising taking part in sessions (step ii) and understanding (step iii), or with the number of concepts, which could hinder application (step iv) through difficulties retaining and recalling ideas. This account also highlighted the importance of access to resources between sessions (b/f 10), which may support understanding and processing (b/f 13).

“*When she’s explaining and long words and that, I’m not really understanding and you constantly have to stop the group and say, oh well what does that mean? … … And others in the group were not sure as well. … … I’d rather it be explained a little bit more, cause obviously I was not really understanding as much, but I didn’t want to stop the group and feel stupid. … … Obviously you can’t look back onto the video and remember*.” (D11; 6+1)

**Co-produced lived experience panel recommendation**: Increase infant-age-specific examples (including in videos), anticipate potential distress regarding “timing,” consider whether accessing the programme with a very young baby (particularly a first child) may be too soon, offer refresher sessions, and improve access to materials outside sessions.

#### 3.3.2 Facilitator and co-facilitator role

Facilitators were the most highly rated aspect: 76% (125/165) rated them “*very helpful*” and 19% (32/165) “*helpful*.” Their empathy, pacing, and clarity supported taking part (step ii) and understanding (step iii), with downstream implications for application (step iv) and feedback loops supporting continued attendance (step i). This format area was closely linked to multiple perspectives (b/f 11), the ability to speak freely or be quiet freely (b/f 12), and flexibility during sessions (b/f 8). Facilitators also supported attendance and taking part by helping parents navigate I. T. challenges (b/f 5) and access COS-P resources (b/f 10).

“*They gave us time to process questions, gave personal experiences & reassured us along the way*.” (E3; 10+0)

“*She was helpful in making sure we understood what was being said*.” (A1; 9+1)

“*I felt really comfortable, which I think, at that point, it was quite unusual for me. … … I did speak very freely and I never felt any judgement or like I was being stupid, which sometimes I would worry before I would say something, so I’d start it off by saying, like, oh, this might sounds stupid but, and it would always be met with, that’s not stupid and, you know, she would always give more examples of different things to just make you feel reassured that the things that you do say are perfectly normal and that, you know, people do relate and that it fits into other circumstances*.” (F34; 5+0; 9 sessions offered)

Criticism of practitioners was uncommon and tended to relate to examples not being sufficiently accessible (affecting understanding, step iii) or to ruptures in group dynamics not being fully addressed (b/f 12; affecting taking part, step ii).

“*When questions were asked responses didn’t deviate much from the wording used within videos*.” (G39; 7+3)

Practitioners described dilemmas around adherence to the script and using personal examples, and also noted increasing confidence across groups, supported by coaching. The co-facilitator role varied across sites but included supporting access through practical tasks (e.g., managing technical issues affecting attendance and taking part, b/f 5), supporting group dynamics (e.g., b/f 12), and contributing additional perspectives, particularly in small groups (b/f 11).

**Co-produced lived experience panel recommendation**: Attend to ruptures (especially online), clarify co-facilitator roles, support practitioner confidence in adapting pace and examples, and consider whether—with appropriate support—peer-support workers could contribute as “bridges” between practitioners and group members by bringing additional lived experience perspectives.

#### 3.3.3 Group format (not one-to-one)

Almost 60% (97/165) found being in a group “*very helpful*” and a further 30% (50/165) “*helpful.*” Groups normalised experiences and supported taking part (step ii) and understanding (step iii) through multiple perspectives (b/f 11) and encouragement to speak freely (b/f 12). However, some parents found groups challenging when they were very small or when contributions were uneven, undermining taking part (step ii).

“*Really helpful to share my experiences and learn or help others*.” (B22; 10+0)

“*I didn’t feel able to open up with the group as not all the other mums were honest*.” (H20; 10+0)

Some parents valued opportunities to socialise and connect with other group members during or outside sessions, describing mutual support that could strengthen continued attendance (step i; feedback loop).

**Co-produced lived experience panel recommendation**: Avoid very small groups; support informal “chit chat” online; use pre-group meetings to set expectations; consider peer-support workers to strengthen psychological safety.

#### 3.3.4 Group size

Nearly one in five respondents (18.2%, 30/165) experienced the group size as “*too small*”; all were in groups with four or fewer completers. Groups sometimes ran with fewer members than planned; practitioners linked this primarily to trial scheduling constraints rather than the programme itself. Small groups could increase pressure to speak, while larger groups could reduce opportunities to share (b/f 12), sometimes constraining taking part (step ii) and overall experience. Group size also shaped exposure to multiple perspectives (b/f 11), which could support understanding (step iii).

“*Questioning was quite intense at times and I felt under pressure to input*.” (F31; 10+0)

“*Not everyone in my group contributed therefore I felt more pressure often to speak even when the subject matter was a bit difficult for me*.” (H20; 10+0)

“*It would have been beneficial to have a slightly larger group for a wider range of experiences*.” (H11; 8+2)

**Co-produced lived experience panel recommendation**: Avoid running very small groups; consider delaying start until more parents can join; use co-facilitators/peer-support roles to add perspectives when numbers are low.

#### 3.3.5 Session structure: length and pacing

Nearly a quarter of parents (23.0%, 38/165) found the session length “*too long*.” Length and pacing interacted with childcare (b/f 1, 2), time commitment (b/f 7), physical comfort (b/f 9), ability to speak freely or be quiet freely (b/f 12), and concentration and processing demands (b/f 13). Variation in site-based break practices shaped taking part (step ii) and understanding (step iii). Home contextual factors (e.g., competing demands on attention) could place additional challenge on cognitive load and disrupt psychological presence, reducing taking part (step ii) and understanding (step iii).

“*It was harder to stay present for the whole [duration] as I needed to prioritise [baby]*.” (A37; 9+0)

“*Sometimes, despite a break, we needed longer breaks… dealing with baby and thinking about difficult things*.” (I35; 8+1)

“*About right to manage my time with my baby but we often didn't get to complete the topics because of people sharing stories and experiences*.” (G30; 10+0)

**Co-produced lived experience panel recommendation**: Build in scheduled breaks alongside responsive breaks; acknowledge emotional intensity and parents’ competing demands (including childcare).

#### 3.3.6 Modality (online, in-person or mix)

Preferences were mixed: 43.0% (71/165) preferred mixed delivery and 20.6% (34/165) preferred fully online. Online delivery supported attendance (step i) by reducing childcare barriers (b/f 1, 2) and removing travel requirements (b/f 3) and travel time, particularly when travelling with a baby (b/f 7). Contextual factors included travel-related difficulties linked to birth injuries or social anxiety.

“*I would not have gone if it was in person… I wouldn’t have been able to get myself in the door because of my anxiety*.” (A33; 6+1)

For some, online delivery facilitated attendance but reduced taking part (step ii) and onward steps, due to home-environment distraction (b/f 6), increased concentration and processing demands (b/f 13), reduced ability to read social cues (b/f 12), and fewer opportunities to observe parent-infant interactions compared with in-person delivery (b/f 11). Similarly, taking part (step ii) was sometimes compromised by I. T. issues (b/f 5), including those relating to connectivity and being able to make timely contributions.

“*Online can be difficult… I was sometimes waiting with my hand up and the moment had passed*.” (F26; 10+0)

For others, online delivery supported access across steps, for example through increased comfort associated for them and their babies through being in their home environment (b/f 6) and the practical digital features (b/f 5). These parents described how muting, camera-off options, and being in a familiar space enabled taking part (step ii) through supporting psychological safety and reducing emotional load, particularly when discussing distressing content:

“*It was useful to be able to turn things on and off. [Baby] didn't necessarily get to hear [the group] and I could talk to and sing to [baby] without everybody having to hear me trying to do that, and that I think was really good. … … my mute is useful. And I also found it quite helpful to be able to be in my own sort of safe space. So emotionally I knew that if I were to find things hard then it would be alright because I was at home*.” (F17; 10+0)

“*Yeah, obviously I got very emotional, very upset. I guess, the good thing about it being online is I can mute and turn off the camera, take that breather if I need to. So, that’s, kind of, the positive side of being behind a screen is I can step away for a few minutes if I need to*.” (J15; 5+0)

Some comments indicated difficulty taking part from home (b/f 6), including feeling less supported or more distressed when “*alone with baby*”, could impede taking part (step ii), understanding (step iii), and continued attendance (step i; feedback loop).

“*Really struggled with online and found it very triggering with a baby while alone. Face to face would have provided support when needed*” (A39; 7+2)

Some parents felt that although leaving home posed barriers, doing so could reduce loneliness and yield benefits beyond the programme, including social opportunities for parents and babies, with potential feedback effects on attendance (step i).

“*I don’t really like going into groups where I don’t know people, but being in that situation and attending that group … sometimes I think, like new mums, they do feel lonely … …So having other mums that are feeling the same, I think it does sort of give people a bit more of a boost*.” (C33; 5+5)

Online delivery could also reduce practitioners’ ability to manage the group environment and enforce expectations about who else was present.

“*At one point someone was doing it in a room with their husband in the background, and that made me feel quite uncomfortable. … Maybe just a reminder of ‘this is the process’ or ‘this is how we do things’, might have prompted them*.” (A39; 7+2)

“*There was one parent whose … middle child … wasn't at an age where she was at nursery, so she was kind of milling about with the partner … so would often appear on the screen. … [That] I could have controlled a bit more if we’d been in the room as opposed to when people are in their own environments. And we did talk right at the beginning about, you know, keeping screens blurred in the background and trying to keep distractions to a minimum, but this is real life, isn’t it?*” (PRAC09)

**Co-produced lived experience panel recommendation**: Recognise that access includes taking part, not only attendance; offer modality options (online, in-person, mixed) where feasible to maximise choice; consider what support is available to parents outside sessions.

#### 3.3.7 Presence of babies

Just under half (46.1%, 76/165) preferred babies to be present for all sessions, 21.8% (36/165) for selected sessions, and 13.3% (22/165) for none. Allowing babies supported attendance (step i) by removing childcare barriers (b/f 1). Comments indicated that, without this option, many parents would have been unable to attend, preventing access entirely. Family contextual factors (e.g., feeding, anxiety, routines) also shaped preferences. As with modality, this feature facilitated attendance for many but introduced barriers elsewhere in the pathway. For example, distraction and increased processing demands (b/f 13), and reduced ability to speak freely (b/f 12), could limit taking part (step ii) and hinder understanding (step iii). These comments usually related to parents’ own babies, but some described distraction related to other participants’ babies.

“*I would not have been able to attend if I couldn’t have had my baby with me… though she did make it harder to focus*.” (C23; 9+0)

Concerns about emotional expression in front of the baby highlighted privacy-related constraints on taking part (step ii).

“*I can understand why women might not want to bring a baby and talk about difficulties in front of the baby, even though perhaps the baby might not understand, but some women might not want to get really upset in front of their baby, or, you know, say things that are quite negative thoughts about the baby, whilst the baby’s there as well. So it does make me think, how open and honest someone can be in a group, where the baby’s then present?*” (PRAC11)

Some parents and practitioners suggested that babies’ presence (own or others’) could support understanding (step iii) by providing live examples that enriched perspectives (b/f 11) and supported application (step iv), although this was not without tension.

“*We're asking them to be monitoring our baby's communication and then we're distracting them with videos on how to do that! And they then can’t attend to their baby!*” (PRAC05)

**Co-produced lived experience panel recommendation**: Recognise that access includes taking part, not only attendance; ensure childcare options support genuine choice; maintain flexibility around camera-off moments and breaks.

#### 3.3.8 Presence of older children

Most parents preferred older children not to attend, primarily due to concerns about parents’ ability to speak freely (b/f 12) constraining taking part (step ii) with some questioning the appropriateness given older children’s greater language and understanding. Family composition and childcare access shaped preferences, including the availability of other adults to care for older children, and the presence of pre-school-aged children. Preferring older children to be present was largely practical (e.g., making attendance possible (step i) where no other childcare was unavailable); however, some parents also felt that older children provided live examples of COS-P concepts thereby supporting understanding (step iii).

“*I would feel uncomfortable having older children present who could remember and repeat discussions*.” (G37; 7.5+1.5)

“*I would like it if we could include older children… we could only see the coming ins and goings in our older children*.” (B37; 10+0)

**Co-produced lived experience panel recommendation**: Consider support for childcare options for older children.

#### 3.3.9 Presence of parenting partners or other adults

Participants expressed highly mixed preferences regarding the presence of someone who supports them with parenting (Table 2), with comments also noting that preferences may vary by session topic. The primary concern was that having another adult present who was not themselves under the care of the service would inhibit disclosure by reducing the ability to speak freely (b/f 12). This was raised most often in relation to participants’ own partners or supporters, but a few comments also highlighted discomfort with the presence of other group members’ partners or supporters. Parents valued a “*safe*” and “*private*” space—a “*space for us*”—where “*us*” referred specifically to birthing parents (typically women) experiencing PMH difficulties.

“*It's easier to be open and honest with strangers in the same boat. Not sure how I would have felt to have other people there as other people’s support*.” (I13; 9+1)

These concerns sometimes extended to discussions of participants’ own experiences of being parented, for example if the group member’s own parent or parent were to accompany them. This underscores how group composition shapes perceived privacy and openness, affecting taking part (step ii).

“*I felt that I could be more honest, particularly about my childhood and how that had influence my parenting, because I was talking to people that I didn’t know*.” (A43; 9+0)

Some parents did not want other adults present because they preferred to process content privately before sharing their understanding with a partner, co-parent or other family member. This shows how processing privately (within a group but away from own family members) may support understanding (step iii) before shared application (step iv).

“*I was able to concentrate on my own thoughts and feelings before sharing my understanding with my partner*.” (B16; 10+0)

Parents who preferred, or were unsure about, other adults attending identified potential benefits for birthing parents, the other adults themselves, babies, and other children in the family. Perceived advantages included improving partners’ understanding of the difficulties affecting birthing parents (including in the context of PMH difficulties and trauma), supporting shared responsibility for parenting with partners or co-parents, and creating a shared understanding that could support joint application of learning (step iv). These accounts foregrounded access as a family-level process rather than solely an individual or dyadic (parent-infant) process.

“*Then we could be using the skills together and supporting each other whilst putting it in place*.” (H15; 6+4)

“*I feel, like, it could have been really good for him to, you know, have [it] as well because we don’t agree on how we both parent*.” (A36; 7+3)

Although views on the acceptability of parenting partners attending were polarised among parents and practitioners, there was broad support for low-resource forms of involvement that would not compromise openness within group discussions. Suggestions included providing an additional, more detailed copy of the workbook and enabling access to videos outside sessions. A minority also noted practical constraints on involving parenting partners, for example where another adult needed to care for the baby and/or older children during sessions to support the group member’s access, or where work commitments prevented attendance.

“*I think he read some notes after we broke up … ‘Oh, I had no idea.’ And I was like, well a bit late*.” (F29; 8+1)

“*The workbook is really great … I was able to use it also with my husband … [to] have the same understanding on how to handle things with the kids. So, I had to go through the booklet twice*.” (G22; 9+1)

“*Mum had a look through it and couldn’t make any sense of it. … The workbook isn’t a standalone document*.” (F15; 9+1)

During interviews, many parent participants described ways in which their partners supported their understanding and application of learning, despite being unable to attend (step i) or take part (step ii) in sessions. Such support could also strengthen feedback loops by enabling continued attendance (step i) and sustained engagement beyond the programme. At the same time, some parents did not have supportive others available to them (in COS-P terms, to “*fill their cup*” or “*be their hands*”), highlighting the importance of context.

“*When I am not having a good day, he is able to bring me back to the theory and just to say, ‘Well look at she is now, playing on her own, and you are here talking to me and she’s not looking, and every now and again she does that visual check.’ He’s able to help me, and that is what has helped our family the fact that I have not, even though I’ve done this on my own, I haven’t done it on my own. He has been able to be that earth connection*.” (J17; 9+1)

**Co-produced lived experience panel recommendation**: Offer low-resource ways for parenting partners to access elements of COS-P outside sessions, where birthing parents wish this to happen.

#### 3.3.10 Pre-group sessions (usually one-to-one)

Pre-group sessions were encouraged within the trial but varied in whether they were offered, and in their length (from 5 min to an hour) and format (phone, video, or in-person in a clinical space or at home). Pre-group meetings were able to prepare parents for accessing the group by clarifying what the sessions involve (including initial socialising to content, such as expectations of reflecting on own experiences of being parented), addressing practical concerns in advance (e.g., worries about feeding or changing their baby during sessions; childcare, b/f 1), and helping build trust and familiarity with the practitioner (relational safety; b/f 12). This preparation could support attending the first session (step i) and taking part (step ii), particularly for parents who had not yet received other care within the PMHS.

“*I didn't know, was it going to just be that we were all going to share about how awful we were feeling? … … I just didn't know what it looked like and I found it difficult, that very beginning because I didn't really have anywhere to ask*.” (F26; 10+0)

“*When I spoke to the facilitator, she just explained what would happen on the sessions, how many mums there would be, what format the sessions would be carried out in, and obviously knowing that I’d have [name of baby] when doing the sessions, that was making me feel anxious, so they made it very clear that if we needed to go away and change a nappy, needed to put baby down for a sleep, if we needed to make a bottle and do a feed, if we had to play with baby, anything we had to do, it was fine. And we would have the opportunity if we felt we’d missed anything to go back over it*.” (B34; 7+3)

Pre-group sessions also helped identify contextual constraints on access (particularly those concerning attendance, step i, and taking part, step ii), including practical issues (e.g., venue-related needs, b/f 4; digital confidence for online delivery, b/f 5) and personal factors such as comfort in groups, comfort discussing mental health with others or previous experiences that shaped trust in services.

“*I told her that I had joined some like bereavement groups before that are online and I really struggled with those. So we talked about like the sort of feelings that came up during those support groups and how we can avoid, well, not avoid but like make it more comfortable for me [in COS-P]. Yeah, so she just gave me lots of reassurance and just let me express my concerns*.” (I26; 9+1)

#### 3.3.11 Catch-up sessions

Catch-up sessions were intended to be offered when a participant missed a session, and although offered to most parents in this position, they varied in format (phone or video for one-to-one; one to one or small-group), staffing (lead facilitator vs. co-facilitator), and length/timing (standalone or early join to the next session). Brief recaps within the main sessions were also valued. Variation in format interacted with contextual factors such as parents’ wider commitments, digital access, and comfort with different communication modes.

Catch-ups served multiple purposes. Some focused on key concepts from missed sessions, supported by COS-P resources and workbook materials (b/f 10). These helped parents re-establish understanding (step iii), support application (step iv), and re-engage in group discussions (step ii), particularly when absences were due to competing demands such as health appointments or infant illness (b/f 7), illustrating their role in supporting continued attendance (step i). Parents generally felt catch-ups were sufficient when only one or two sessions had been missed.

“*But because we’d done the catch-up, I knew it all, and I was where everybody else was, and, yeah, it was really nice*.” (B29; 9+1)

However, some noted that catch-ups lacked the reflective depth and multiple perspectives of the group (b/f 11), and were sometimes delivered without the protected processing time (b/f 13), constraining deeper understanding (step iii) and early application (step iv). When catch-ups were missed or conducted in distracted settings, parents described difficulty following group discussions on return:

“*I felt like I should’ve tried harder to get the catch-up session… I couldn’t [join in for the subsequent main session] ‘cause I didn’t know what we were talking about*.” (A33; 6+1)

“*The catch-up session [by phone], I was sat in the car in a hospital car park … … So I think I was equally a bit foggy. But I read through the workbook, I tried to understand it. I wouldn’t say I didn’t understand it, I’m not able to recognise it in [name of baby] as much as the other things*.” (B34; 7+3)

More individualised catch-ups also functioned as broader check-ins, enabling practitioners and parents to identify barriers to continued attendance (step i), explore contextual challenges, and assess whether alternative support within PMHS would be more appropriate. In some cases, these discussions supported collaborative decisions to discontinue, taking account of psychological distress, trauma-related triggers, or difficulties in the parent-infant relationship.

## 4 Discussion

This study is the first to describe barriers and facilitators to parents accessing COS-P within a PMHS context, using a format delivered predominantly online with babies present. The findings extend the COS-P literature and indicate areas where further tailoring may better meet the needs of this clinical population. The paper also offers an empirically grounded framework for conceptualising equitable access to group-based psychological interventions in the perinatal period for individuals and their families, drawing on findings from the COSI trial.

Our findings move beyond a narrow focus on attendance by conceptualising access as a four-step pathway—attend, take part, understand, and apply—characterised by five principles. A key conceptual contribution is that access requires both physical and psychological presence. Psychological access is not visible and cannot be inferred from attendance. Parents may be in attendance but unable to take part or understand for reasons that are not externally observable (e.g., concentration difficulties, processing load, emotional triggers, or discomfort speaking freely). Conversely, some parents may appear “absent” because they choose not to use a camera, speak, or write in the chat, while nonetheless taking part psychologically and understanding the content. Relatedly, intervention-format features intended to facilitate access may unintentionally create barriers.

Considering each component of the intervention format in turn (e.g., modality, group size, session structure, presence of babies) enabled identification of when and how barriers and facilitators were experienced, and how these experiences were shaped by individual, family, and location contexts. The utility of the format areas lies in offering tangible elements that surface access issues and guide practitioners—and parents and their families—towards potential solutions. Similarly, the empirically derived contextual factors provide prompts for designing and reviewing delivery, addressing inequities in access, and supporting structured conversations with parents.

### 4.1 Contributions to COS-P literature

A distinctive contribution of our study concerns the fit of COS-P for parents of very young infants. Parents frequently emphasised the importance of developmentally attuned materials, noting gaps in examples relevant to younger babies. Some also suggested “top up” or follow up sessions to support consolidation and continued application, echoing Gilhooly’s (2018) finding that parents valued additional opportunities for reinforcement. For a perinatal population, such top ups may be particularly beneficial given the rapid developmental changes in infant communication across the first year. These COS-P specific insights point to the value of age relevant materials and ongoing support to sustain engagement, understanding, and application beyond the core sessions.

Consistent with research in other populations (e.g., early parenting services, Maxwell et al., 2021), COS-P was largely experienced as accessible across our datasets, reflected in survey ratings, open-ended comments, and qualitative narratives. Our findings align with Maxwell et al.’s (2021) emphasis on “making complex theory accessible”, while also showing instances where parents felt overwhelmed by the volume of concepts or experienced difficulty retaining and applying multiple ideas in real time. This underscores the importance of breaks—not only for physical comfort and attending to babies, but also for cognitive processing and emotional regulation—to sustain taking part and understanding, particularly in online delivery. Break practices varied across sites, highlighting a need for clearer guidance in training, especially for online groups.

Even where strategies were in place to promote access, there were instances suggesting that a group setting—and the associated pressure to cover required content—may not be appropriate for all parents. This may be particularly salient in this clinical context given the pervasive effects of mental health difficulties and trauma, alongside parenting stress and its impact on maternal neurocognitive functioning (e.g., attentional control, working memory) (Callaghan et al., 2024), and the additional burden of sleep deprivation in the perinatal period. Parents’ and practitioners’ appreciation of the baby-specific edits to the workbook further supports the need for developmental tailoring. Expanding younger baby examples may help achieve three linked aims: sustaining taking part and understanding during sessions, strengthening application between sessions, and enabling parents to involve partners or other caregivers in understanding and applying COS-P learning outside sessions.

### 4.2 Contextual factors shaping access

Our findings indicate that contextual factors are fundamental to meaningful access and help explain why the same intervention-format feature can be experienced differently across families. These findings also highlight that attendance is necessary but not sufficient; meaningful access depends on the extent to which parents are able to take part, process, and apply COS-P learning across pathway steps. Contextual factors shaped how barriers and facilitators were activated, which pathway steps were most affected, and how parents experienced the balance of facilitating and constraining influences across steps.

Material (socioeconomic) resources can affect both attendance and taking part. This is central to addressing inequalities in access, including for marginalised groups; for example, parents from racially minoritised backgrounds and lone parents, who are more likely to experience socioeconomic disadvantage. For in-person provision, material constraints include transport cost and time, and—for those reliant on public transport—coverage and reliability, which may disproportionately affect those in rural or geographically isolated communities (Ferris-Day et al., 2021). For remote delivery, material resources include access to an appropriate device (including adequate screen size), headphones, data allowances, and stable connectivity. This is consistent with calls to address both digital poverty and data poverty (Watts, 2020), with connectivity problems disproportionately affecting rural and geographically isolated communities and those living in poorer housing. The home environment is also salient and may disproportionately affect families living in constrained conditions, with implications for privacy, competing demands, and the ability to sustain psychological presence.

Individual context relating to communication and cognitive needs can affect all pathway steps. These include factors closely linked to mental health and trauma, alongside language fluency, complexity of ideas, social communication, and processing needs, with neurodiversity potentially relevant across these domains. Autistic women in the perinatal period face additional challenges in comprehension and social interaction, including in group-based contexts (Westgate et al., 2024), strengthening the case for plain-language explanations, careful pacing, and visual scaffolding.

Online group delivery can substantially facilitate attendance. For some parents, benefits extend across the pathway; for others, including those who would otherwise be unable to attend, online delivery may compromise later steps by introducing new challenges. For example, flexibility features (e.g., optional camera use, chat functions) widened access for some but at times generated tensions in group dynamics that practitioners needed to actively bridge.

Family dynamics and preferences shaped access at all pathway steps. Childcare availability for babies and older children, or the option to attend with babies (negating the need for childcare), enabled attendance for some parents while being undesirable for others. Infant age, older children in the family, and the degree of support in a parent’s network influenced parents’ ability to take part, understand and apply COS-P learning. These findings underline the importance of aligning programme timing and tailoring with family context.

### 4.3 The relevance of systemic thinking for understanding and supporting access

This analysis reinforces the importance of systemic thinking in perinatal mental healthcare, consistent with policy (NHS England, 2019) and guidance on involving partners and wider family members (Darwin et al., 2021). Conceptualising access as relational and contextual extends these principles to group-based and increasingly online interventions.

A systemic lens reframes access as co-constructed within relational systems—where relevant involving the infant, other children, partner, and wider family—and shaped by the environments in which support occurs. Applying systemic principles can (a) support the birthing parent’s ability to attend and take part; (b) strengthen individual understanding and application during and between sessions; and (c) promote shared understanding and joint application across the family.

By considering who access is for, who can influence it, and where and when access occurs, our framework extends family-inclusive thinking to the broader range of environments in which PMHS support individuals, dyads, and families. These include settings where practitioners exert greater influence (e.g., community venues) and those where they have less influence (e.g., home environments), as well as the newer territory of online spaces with distinct affordances and constraints (e.g., camera use, blurred backgrounds, mute functions).

### 4.4 Implications for practitioners and services

We propose using the framework in practice to make access more equitable and meaningful. Specifically, the intervention-format areas and contextual factors (individual, family, location) can be used as prompts to anticipate pathway impacts (attend, take part, understand, apply), identify likely opposing effects, and co-design counterbalancing supports with parents and families. This could be embedded in treatment planning, pre-group meetings, catch-up sessions, and other clinical review points.

While services are under pressure to deliver cost savings, reducing unfair and avoidable differences in access requires targeted investment in material resources. Informed by our analysis and co-produced recommendations, we suggest that a minimum specification for group interventions for services and commissioners should include:

- offering a choice of modality where feasible
- providing access to intervention resources between sessions (e.g., videos and written materials)
- establishing explicit norms about privacy that are workable across different home environments, with facilitators trained to address and support this
- incorporating planned breaks
- providing devices (e.g., through a device library), headphones, and data
- selecting venues for in-person delivery that have strong transport links and ensuring transport costs are covered, as appropriate; and
- providing structures outside of group sessions (i.e., pre-group meetings and catch-up sessions) to support access (recognising that these also have access considerations).

We encourage services to support family-inclusive approaches by using adjacent forms of communication or involvement that do not replace or interfere with what happens in the group, ensuring that psychological safety within the group is not compromised. Examples include shareable resources where appropriate to family context (e.g., brief orientation materials, access to session resources within licencing, post-session summaries) or offering a separate opportunity for a parent and their partner or other support person to meet with a practitioner or co-facilitator.

### 4.5 Implications for future work

Practice and research often default to attendance as the primary access metric; our findings suggest this constrains the ability to identify and address disparities in meaningful access. By framing access as a pathway, researchers may better identify which parents, babies, and families are most affected, and how this relates to outcomes.

We propose using the framework prospectively as a planning and evaluation tool to monitor access at each pathway step, identify opposing effects, and assess whether adaptations reduce inequities. Priority initiatives to design and evaluate include the impact on access patterns across diverse communities of resource decisions (e.g., crèche provision, transport reimbursement, data allowances, device access), strategies to involve other adults (e.g., other caregivers or a parent’s key support person), and different settings (community venues, home environments, virtual spaces).

### 4.6 Strengths and limitations

Strengths include multi-site delivery (supporting learning across variation in practice while holding core COS-P materials constant), a robust survey response across sites, inclusion of both completers and non-completers (with missed sessions offering insight into attendance barriers), and analysis involving lived experience co-researchers and practitioners, supporting interpretive depth and contextual sensitivity. Although delivery was predominantly online, many participants also had some in-person exposure, enabling comparative reflections across modalities.

A key limitation is the limited racial and ethnic diversity in the wider trial population. Sites suggested that this pattern may reflect wider service-level access disparities rather than local population composition, aligning with previously reported inequalities in access (Jankovic et al., 2020). While members of the lived experience panel delivered workshops on inclusive research recruitment, these focused on who was already accessing services rather than shifting access into PMHS itself. A small number of COS-P parents in the COSI trial were supported by interpreters; however, we did not collect data on access experiences related to interpretation, limiting learning in this important area. A further limitation is that our qualitative analysis centred on participants with at least some exposure to COS-P. It is important to note that non-starting can itself reflect access barriers, with non-starters’ trial data citing reasons related to practical constraints (most commonly being unavailable at the session times and lack of childcare for older children), feeling uncomfortable in group settings, or being too unwell to take part. Although detailed insights were not available for this group, the limited information available shows clear parallels with the access issues identified in this paper.

Nonetheless, the framework remains valuable for equity work because it foregrounds elements (e.g., material resources, privacy, language demands, and cognitive load) that plausibly underpin disparities regardless of sample diversity, and which may operate both for parents who start COS-P and for those unable to start at all. These factors may be particularly salient for racially and ethnically marginalised parents.

## 5 Conclusion

This paper identifies multiple barriers and facilitators to accessing COS-P in the context of PMH difficulties and a group format delivered predominantly online. By locating these within an access framework, the relevance of the findings extends beyond COS-P, offering transferable learning on how to conceptualise and address access to group psychological interventions in the perinatal period. Appreciating the complexity of accessibility may help widen access in ways that are more meaningful to individuals and families, including through systemic consideration of who access is for, where access takes place, and who can influence access. This is necessary to ensure that apparently convenient and less resource-intensive ways of working do not inadvertently deepen inequalities.

## Data Availability

The datasets presented in this article are not readily available because the datasets generated and/or analysed during the current study are not publicly available. As specified in the protocol, while names of places and people have been removed, the combination of contextual information provided by participants could compromise anonymity if data were made available in full. Requests to access the datasets should be directed to z.darwin@hud.ac.uk.
