# Supplementary material for: Widening access to perinatal mental health group interventions: learning from a trial of the Circle of Security-Parenting programme in England
Source: Front Psychol. 2026 Mar 26;17:1802417. doi: 10.3389/fpsyg.2026.1802417 (PMC13063578; doi:10.3389/fpsyg.2026.1802417)
Supplement: Supplementary file 1 [file Table_1.DOCX]

**Supplementary File 1 Mapping exercise from a qualitative team meeting, involving co-researchers with lived experience**

This mapping exercise from an analysis meeting with lived‑experience co‑researchers, identifies factors that may act as barriers or facilitators for each format area and how these may operate differently or additively across the access pathway (attend, take part, understand, apply). It illustrates that some factors connected broadly across pathway steps and intervention format areas (e.g., b/f 12 and 13), whereas others were more specific (e.g., b/f 3 and 4 relating mainly to attendance). It is not intended offer an exhaustive list.
