# Supplementary material for: Widening access to perinatal mental health group interventions: learning from a trial of the Circle of Security-Parenting programme in England
Source: Front Psychol. 2026 Mar 26;17:1802417. doi: 10.3389/fpsyg.2026.1802417 (PMC13063578; doi:10.3389/fpsyg.2026.1802417)
Supplement: Supplementary file 2 [file Table_2.docx]

### Supplementary File 2 Examples from qualitative team meeting discussions of how individual and family context factors may relate to access across the four-step pathway and to specific barriers and facilitators

| **Individual and family contextual factors** | **Examples of how these may relate to steps on the access pathway (i.e. attend, take part, understand, apply) and factors that are barriers and facilitators (b/f)** |
| --- | --- |
| **1 baby’s age** (e.g. ease of attending and taking part at different developmental stages, relatability of concepts) | -May find it easier/harder to attend and to take part, varying with baby’s needs (e.g. feeding, mobility). This also links to who the access is for (i.e. may not attend alone) and where the access takes place. For example, home environment (b/f 6) may enable attending and taking part through offering familiar environment with own toys; the comfort with using childcare creche facilities for in-person may vary with baby’s age.  -May find it harder to understand and applying the intervention learning if baby is very young (e.g. less easy to ‘see the circle’ with own baby) so may particularly value multiple perspectives in the group (b/f 11) to learn from examples with older babies in the group, or older children in the families. |
| **2 family composition** relating to children (e.g. older children, perinatal loss) | -May find it harder to join from home environment (b/f 6) where older children are present yet may be unable to attend in-person without provision of childcare for older children (b/f 2).  -May find it easier to understand and apply intervention through having experiences with older children in the family, including own children but also wider family members (e.g. nieces, nephews, younger siblings; b/f 11).  -May find it harder to apply intervention as planned because of demands placed when parenting multiple children (i.e. children of different ages or multiple birth); some comments indicating this is not fully accounted for within intervention.  -May find it harder to take part and understand in the context of perinatal loss where distress compromises ability to concentrate or process (b/f 13), or discomfort with voicing own experiences (due to own or anticipating others’ distress) compromises ability to speak freely (b/f 12). |
| **3 own mental health and trauma** (e.g. flashbacks, concentration, memory, not wanting to be seen, exhaustion, inpatient admission) | -May find intervention easier/harder to access (all four steps) depending on own current mental health (e.g. if experiencing flashbacks, struggling with mood or concentration); for example, all steps may be influenced by ability to concentrate and ability to process (b/f 13).  -May find it harder to attend and take part due to greater demands relating to travel (b/f 3), physical comfort (b/f 9) and the required time commitment (b/f 7) when struggling with mental health and trauma, including exhaustion.  -May find it harder to apply intervention because of challenges with emotion regulation, linked to mental health and trauma. |
| **4 own physical health** (general short and long-term; postpartum recovery) | -May find it harder to attend or take part when physical health limits mobility or comfort, interacting with transport (b/f 3), physical comfort (b/f 9), and time commitment (b/f 7).  -May find it harder to take part where pain, sleep disturbance, or birth injuries/recovery limit sustained focus or sitting tolerance (b/f 9).  -May find it harder to understand and apply, linked to symptoms impacting processing load (b/f 13). |
| **5 wider circumstances** (e.g. relationship breakdown,  bereavement, financial circumstances, housing) | -May find intervention harder/easier to access (all four steps) depending on wider life circumstances; for example, links with home environment (b/f 6) or impacting attending through schedule clashes (time commitment with return to work or moving home, b/f 7).  -May find emotional strain affects ability to take part or understand reflective content, shaping psychological access (b/f 12), or that relationship breakdown with a baby’s co-parent may enable understanding and applying the learning (b/f 11).  -May experience shifting priorities that influence readiness to attend or apply learning. |
| **6** ‘**culture**’ (e.g. sociocultural or wider family expectations for who is involved with parenting, or how to interact with babies) | -May interpret intervention content through wider cultural norms or family expectations around parenting roles or emotional expression, affecting comfort speaking freely (b/f 12) or interactions with different perspectives (b/f 11).  -May experience mismatch between intervention ideas and held beliefs about infant needs, influencing taking part, understanding and applying. |
| **7 learning styles/preferences** (e.g. visual learning, discussion-based learning) | -May find some session elements more or less accessible depending on preferences for visual materials (b/f 10), discussion (b/f 12), or pacing (b/f 8), with implications for taking part and understanding. |
| **8 distinct cognitive needs** (e.g. memory difficulties, processing speed, struggling with complex language) | -May find intervention harder to take part in and understand and apply (with feedback loops for subsequent attendance) in the context of distinct cognitive needs that may relate to ability to concentrate and process (b/f 13) when delivered in a group context. Although there may be some flexibility in sessions (b/f 8) relating to pacing, there will be limits to this when managing the needs of all group members, and potentially may be more difficult for the facilitator to be aware of this when working online. |
| **9 distinct communication needs** (language and social) (e.g. non-English speaking background, hearing loss, neurodiversity) | -May find intervention harder to take part in and understand and apply (with feedback loops for subsequent attendance) in the context of distinct communication needs that may be supported or hindered with different technologies (b/f 5) and may relate to ability to process (b/f 13) and contribute to the content and conversations (b/f 12). Although there may be some flexibility in sessions (b/f 8) relating to pacing, there will be limits to this when managing the needs of all group members, and potentially may be more difficult for the facilitator to be aware of this when working online. |
| **10 (dis)trust of services** (e.g. comfort in sharing with practitioners that could link to ability to speak freely) | -May hesitate to speak freely due to concerns about judgement or safeguarding, reducing ability to speak freely (b/f 12), compromising taking part and onwards steps. -May require extended time to build trust, influencing early attendance and comfort with taking part. |
| **11 comfort with perceived purpose of intervention** (e.g. parenting vs. mental health vs. relationships; under scrutiny) | -May find it harder/easier to attend and take part if perceiving that intervention has been offered due to an identified need in the parent-infant relationship  -May find it harder to take part if perceiving that contributions may be scrutinised by others (for example possible pressures relating to social care involvement), linked to ability to speak freely and ability to be quiet freely (b/f 12). |
| **12 comfort with groups** | -May find group settings anxiety-provoking, affecting attending or taking part.  -May take longer to feel safe contributing (b/f 12), shaping taking part.  -May experience increased comfort over time as relational safety grows, improving access across steps; highlighting that contextual factors need not be static or fixed. |
| **13 comfort discussing mental health with others** | -May struggle to take part when discomfort discussing mental health affects ability to speak freely (b/f 12).  -May find emotionally sensitive content harder to process (b/f 13), affecting understanding.  -May become more comfortable over time as group familiarity increases. -Note in this dataset, comfort with groups was spoken about more commonly than discussing mental health with others, and closely linked. |
| **14 comfort with being apart from baby** | -May not feel comfortable being apart from baby even if there is access to childcare (either in own network or through the service), however may find having baby present supports comfort but introduces distraction (b/f 13), impacting subsequent access steps. |
| **15 own professional background** (e.g. use of online working, knowledge of child development) | -May work in a particular role (e.g. health visiting, mental health, nursery nursing, early years, social work) and draw on existing knowledge, influencing ease of understanding in relation to materials (b/f 10).  -May experience tension between professional identity and group member role, affecting comfort speaking (b/f 12). |
| **16 other care before/alongside intervention** (e.g. may impact ability to concentrate and process, to apply learning) | -May find it harder to work with intervention concepts and emotional intensity (b/f 13) if not yet received other care in the service, or not received alongside care to help with managing distress, impacting subsequent attendance.  -May find it difficult to attend if different aspects of care place high demand on time (b/f 7).  -May encounter synergy, overlap or tension between approaches that shape understanding and application. |
| **17 support from (parenting) partner/others in own network** (e.g. childcare, transport, device, talking after sessions, sharing learning, consolidating learning, support with applying) | -May require practical assistance to attend the group (e.g. looking after baby/children during intervention b/f 1, b/f 2; giving a lift to in-person session b/f 3; borrowing laptop b/f 5).  -May receive support outside of sessions from people in own network to feel safe enough to continue to attend future sessions, which may be closely linked to own context concerning mental health and trauma.  -May strengthen understanding when able to discuss with others outside of sessions (b/f 11).  -May find intervention easier to apply when supported emotionally or practically by others outside of sessions, or harder to apply when parenting without another supportive adult. |
